# Effect of KIOM-79 on Diabetes-Induced Myocardial Fibrosis in Zucker Diabetic Fatty Rats

**DOI:** 10.1155/2013/547653

**Published:** 2013-11-13

**Authors:** Junghyun Kim, Eunjin Sohn, Chan-Sik Kim, Yun Mi Lee, Kyuhyung Jo, Jin Sook Kim

**Affiliations:** Korean Medicine Based Herbal Drug Development Group, Herbal Medicine Research Division, Korea Institute of Oriental Medicine, 1672 Yuseongdae-ro, Yuseong-gu, Daejeon 305-811, Republic of Korea

## Abstract

KIOM-79, a herbal mixture of parched Puerariae radix, gingered Magnoliae cortex, Glycyrrhizae radix, and Euphorbiae radix, has a strong inhibitory effect on advanced glycation end products (AGEs) formation. We investigated the beneficial effects of KIOM-79 on cardiac fibrosis in Zucker diabetic fatty (ZDF) rats. KIOM-79 (50 or 500 mg/kg/day) was orally administered for 13 weeks. AGEs formation and collagen expression in the myocardium were assessed by immunohistochemistry. The expression levels of the receptor for AGEs (RAGE), transforming growth factor-**β**1 (TGF-**β**1), collagen IV, fibronectin, urotensin II, and urotensin II receptor were examined in the myocardial tissue of ZDF rats. KIOM-79 treatment at 500 mg/kg inhibited the accumulation of AGEs, reduced RAGE mRNA and protein expression, and reduced the upregulation of cardiac fibrogenic factors, such as fibronectin and collagen IV, in heart of ZDF rats. Additionally, KIOM-79 ameliorated urotensin II/receptor gene expression in the cardiac tissue of ZDF rats. Our findings indicate that KIOM-79 diminishes cardiac fibrosis in ZDF rats by preventing AGEs accumulation and RAGE overexpression and by modulating the cardiac urotensin II/receptor pathway, which decreases the amount of profibrotic factors, such as TGF-**β**1, fibronectin, and collagen in cardiac tissue.

## 1. Introduction

Diabetic cardiomyopathy is characterized by myocardial fibrosis, which leads to decreased elasticity and impaired contractile function of the heart. The link between hyperglycemia and the development of diabetic cardiomyopathy involves the accumulation of advanced glycation end products (AGEs) in cardiac tissue. Within the cells, AGEs and their precursors modify molecules and produce irreversible cross-linkages between extracellular matrix proteins [1, 2], which compromises tissue compliance and produces myocardial stiffness [3, 4]. AGEs-mediated modification of matrix proteins disrupts matrix-matrix and matrix-cell interactions, which contributes to their profibrotic properties. AGEs play an important role in cell signaling through their interaction with specific receptors, namely, the receptor for advanced glycation end products (RAGE), which activates adhesion molecules, proinflammatory cytokines, and growth factors, and contributes to the pathogenesis of diabetic complications [5].

Transforming growth factor-*β*1 (TGF-*β*1) is regarded as the main profibrotic factor and upregulation of TGF-*β*1 protein expression was observed in the myocardium of rodents with diabetic cardiomyopathy [6]. Profibrotic factors have been pathogenetically linked to the excessive accumulation of collagenous matrix in a wide range of organ and disease states, including the diabetic heart [7].

Recently, the potent vasoconstrictor peptide urotensin II has emerged as a contributor to the pathology of cardiovascular disease. It has been suggested that urotensin II also plays a role in cardiac fibrosis, and it has been identified within the heart [8], where there is an abundant expression of urotensin II receptor [9]. Recent studies reported that the upregulated expression of urotensin II and its receptor were accompanied with profibrotic factor TGF-*β*1 expression and extracellular matrix accumulation in the kidney and myocardium of diabetic animal models [10–12]. 

KIOM-79 is a new herbal prescription and composed of four medicinal herbs: parched Puerariae Radix, gingered Magnolia Cortex, Glycyrrhiza Radix, and Euphorbia Radix, and each herb has been used in traditional Korean medicine and other countries for a variety of medical purposes, including diabetes [13, 14]. Pueraria radix has potential medicinal benefits in diabetes and cardiovascular disease [15]. Honokiol, a major bioactive compound of *Magnolia officinalis*, has anti-inflammatory and antifibrotic effects [16]. Glycyrrhizin, a major bioactive compound of Glycyrrhizae radix, inhibited hepatic fibrogenesis [17]. Euphorbiae radix has antihyperglycemic [18] and anti-AGEs effects [19]. Our previous studies reported that KIOM-79 has a pharmacological effects on diabetic condition [20–23]. In addition, KIOM-79 prevented S100b-induced TGF-*β*1, fibronectin, and NF-*κ*B expression in mesangial cells cultured under diabetic conditions and the progression of diabetic nephropathy in type 2 diabetic rats [24, 25].

Thus, the aim of the current study is to examine the pharmacological effects of KIOM-79 on diabetic cardiac fibrosis in Zucker diabetic fatty (ZDF) rats, which is a genetic animal model of type 2 diabetes.

## 2. Materals and Methods

### 2.1. KIOM-79 Preparation

The cortex of *Magnolia officinalis* Rehd. et Wils. (Magnoliaceae), radix of *Pueraria lobata *Ohwi (Leguminosae), radix of *Glycyrrhiza uralensis *Fisch (Leguminosae), and radix of *Euphorbia pekinensis *Ruprecht (Euphorbiaceae) were collected from the Gamsuk province in China in 2003 after identification by botanist Professor J. H. Kim (Department of Life Science, Kyungwon University, Korea). All voucher specimens were stored at the herbarium of the Korea Institute of Oriental Medicine (Nos. 1240, 2, 7, and 207, resp.). Magnoliae cortex (100 g) was simmered with 3 g of Zingiberis rhizoma for 60 min. Puerariae radix (100 g) was stir-roasted at 75°C for 45 min; when its surface became yellow with brown spots, it was removed and cooled. Equal amounts of gingered Magnoliae cortex, parched Puerariae radix, Glycyrrhizae radix, and Euphoriae Radix were mixed, pulverized, extracted in 80% EtOH for 1 week at room temperature, concentrated with a rotary evaporator, and lyophilized. The entire procedure was repeated for four times. The quality of KIOM-79 was controlled by HPLC [19].

### 2.2. Animal Treatment

Male Zucker diabetic fatty (*fa/fa*, ZDF) and Zucker lean (*fa/+* or +/+, ZL) rats were obtained at 6 weeks of age from Charles River Laboratory (Wilmington, MA, USA). Rats were allowed free access to water and food. KIOM-79 was dissolved in water and orally administered to the rats for 13 weeks. Animals were divided into four groups: Zucker lean rats (ZL, *n* = 7); Zucker diabetic fatty rats (ZDF, *n* = 7); Zucker diabetic fatty rats treated with KIOM-79-50 (50 mg/kg body weight, ZDF + KIOM-79, *n* = 8); Zucker diabetic fatty rats treated with KIOM-79-500 (500 mg/kg body weight, ZDF + KIOM-79, *n* = 8). The dosage of freeze-dried powder was calculated based on the human equivalent dosage of raw herbs. At the end of experimental period, the body weight and heart weight of the rats were measured. The ZDF rats were anesthetized with diethyl ether, and blood and tissue samples were taken for glucose measurement and histological examination. All experimental protocols involving the use of animals were conducted in accordance with National Institutes of Health (NIH) guidelines and approved by the Committee on Animal Care of our institute. 

### 2.3. Western Blot Analysis

Cardiac tissue (0.1-0.2 g) from the rats was lysed with a homogenizer at 3000 rpm in a solution containing 250 mM sucrose, 1 mM ethylenediaminetetraacetic acid (EDTA), 0.1 mM phenylmethylsulfonyl fluoride (PMSF), and 20 mM potassium phosphate buffer at pH 7.6. Equal amounts of protein (50 *μ*g) were analyzed with immunoblotting techniques with the indicated antibodies. The antibodies used were as follows: TGF-*β*1, RAGE, collagen IV (1 : 1000, Santa Cruz Biotechnology, CA, USA), and *β*-actin (1 : 3000, Sigma, USA). A horseradish peroxidase-conjugated secondary antibody was used and detected with an enhanced chemiluminescence detection system (iNtRON Biotechnology, Korea). Protein expression levels were assessed by analyzing the signal from the PVDF membranes using an image analyzer (Las-3000, Fuji photo, Tokyo, Japan).

### 2.4. RNA Isolation and RT-PCR

Total RNA was isolated using TRIzol reagent (MCRC, Cincinnati, OH, USA) according to the manufacturer's instructions. cDNA was synthesized with 3 *μ*g of RNA using RT-premix (Bioneer, Korea). RNA was reverse transcribed using a Takara PCR Thermal Cycler (Japan). Primer sequences are summarized in Table 1. RT-PCR products were separated by electrophoresis with 1.2% agarose gels containing ethidium bromide (EtBr), and DNA band intensities were quantified using densitometry (Las-3000, Fuji photo, Tokyo, Japan).

### 2.5. Immunohistochemistry

To determine collagen deposition in the cardiac tissue, paraffin-embedded sections were deparaffinized, sectioned, and stained using Masson's trichrome. For AGEs immunohistochemistry, the deparaffinized sections were hydrated and treated with 1% H_2_O_2_ in methanol. Sections were incubated with anti-AGEs antibody (1 : 100, Transgenic Inc. Kobe, Japan) for 2 h at room temperature using a standard manual immunoperoxidase procedure with streptavidin-peroxidase (LSAB 2 kit, Dako, CA, USA). The stained sections were observed using a light microscopy (Olympus BX51, Japan) equipped with an Olympus DP 71 camera. The intensity of the staining tissue sections was analyzed using Image J software (NIH).

### 2.6. Statistical Analysis

Data are expressed as the mean ± S.E.M and analyzed by one-way analysis of variance (ANOVA) followed by Tukey's multiple comparison test or by an unpaired Student's *t-*test using GraphPad *Prism 4.0* software (Graph pad, San Diego, CA, USA). Differences with a value of *P* < 0.05 were considered to be statistically significant.

## 3. Results

### 3.1. Glucose Level, Body Weight, and Organ Weight of Experimental Rats

Table 2 shows the effect of KIOM-79 treatment on the general biochemical parameters of blood glucose levels, body weight, and heart weight. Untreated ZDF rats exhibited markedly increased blood glucose levels compared to control ZL rats (*P* < 0.05) and did not exhibit differences in glucose levels compared to ZDF rats treated with KIOM-79. Body weight gain in untreated ZDF rats was significantly higher than that of control ZL rats. KIOM-79 treatment did not change the body weight of ZDF rats when compared to untreated ZDF rats. There were no significant changes in heart weight in the rats in all groups.

### 3.2. Effect on AGEs Accumulation and RAGE Expression in Heart Tissue

**Figure 1:**
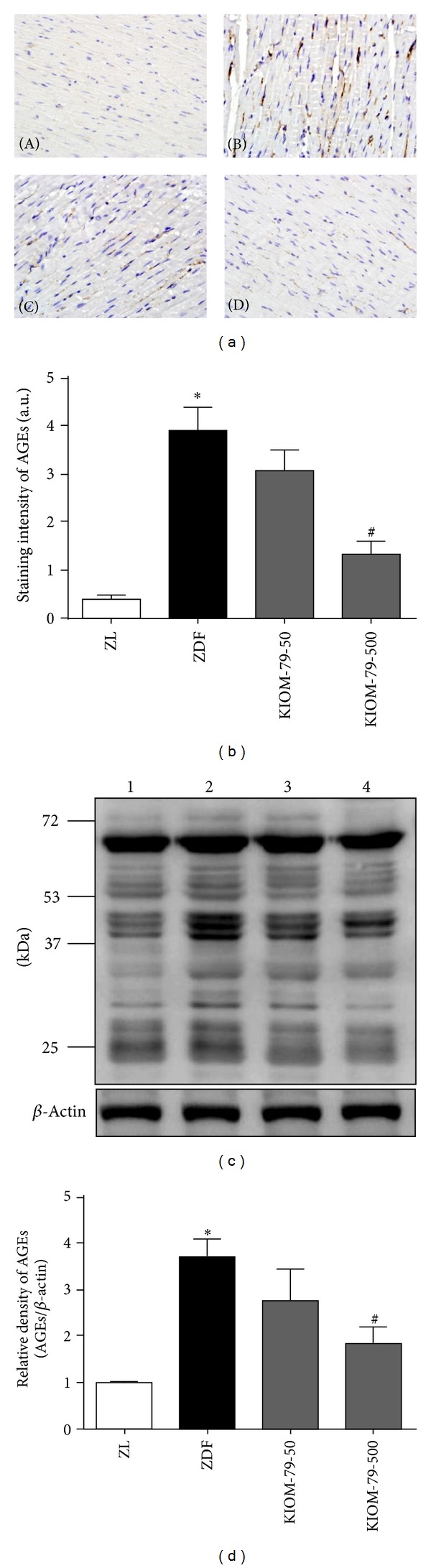
Effect of KIOM-79 treatment on AGEs accumulation in the heart. (a) Representative immunohistochemistry images for AGEs (a) staining in the heart of ZL rats (A), untreated ZDF rats (B), ZDF rats treated with KIOM-79 (50 mg/kg) (C), and ZDF rats treated with KIOM-79 (500 mg/kg) (D). Original magnification: ×400. (b) Quantitative analysis of AGEs stain was calculated. ((c) and (d)) Western blot analysis of AGEs in cardiac tissue. The nonenzymatic reaction of the amino groups of cellular proteins with reducing sugars forms a variety of AGEs. Thus, the multiple bands for advanced glycation adducts were detected. All data were expressed as the mean ± S.E.M. *P < 0.05 compared to ZL rats; ^#^ *P* < 0.05 compared to untreated ZDF rats.

**Figure 2:**
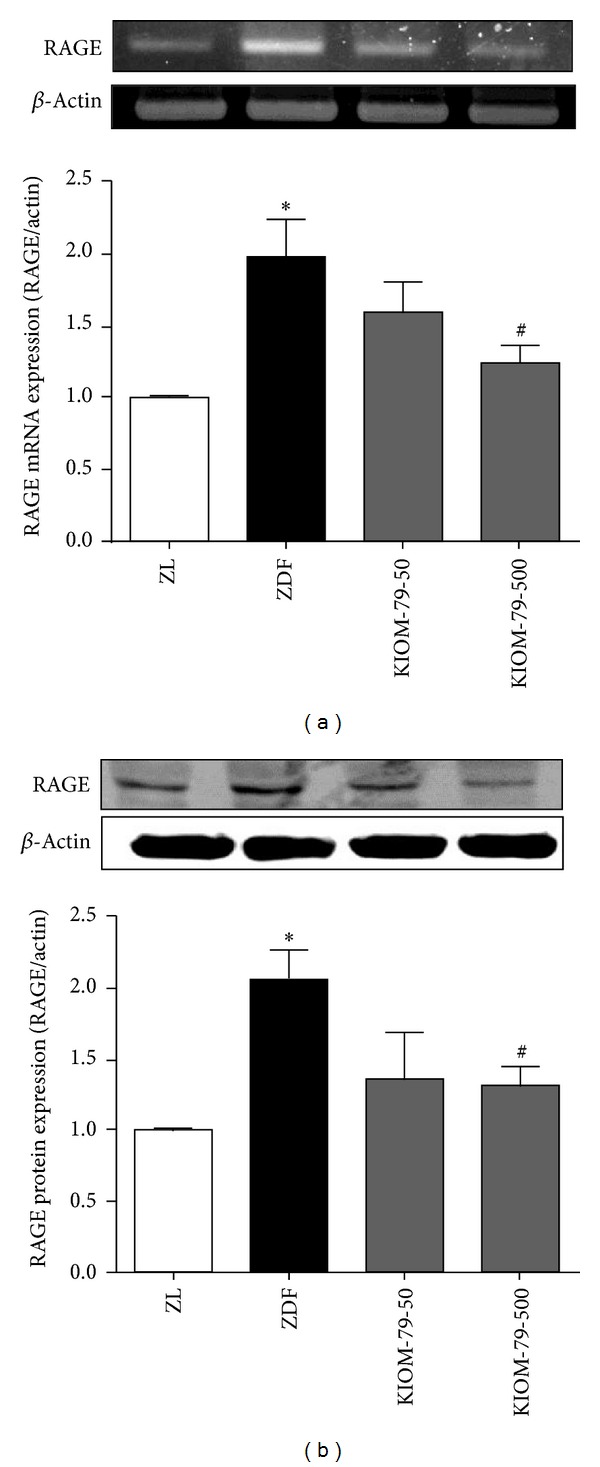
Effect of KIOM-79 treatment on RAGE expression in the heart. RAGE mRNA expression (a) and RAGE protein expression (b) in Zucker lean rat (ZL), Zucker diabetic rat (ZDF), ZDF rat treated with KIOM-79 at 50 mg/kg (KIOM-79-50), and ZDF rat treated with KIOM-79 at 500 mg/kg (KIOM-79-500). All data were expressed as the mean ± S.E.M. *P < 0.05 compared to ZL rats; ^#^ *P* < 0.05 compared to untreated ZDF rats.

Immunohistochemistry was used to determine whether KIOM-79 inhibits AGEs accumulation in the heart. Untreated ZDF rats had significantly higher levels of AGEs accumulation in the heart when compared to control ZL rats (*P* < 0.05). However, KIOM-79 treatment suppressed AGEs accumulation markedly in ZDF rats compared to untreated ZDF rats (*P* < 0.05) (Figures 1(a) and 1(b)). The protein expression of AGEs in cardiac tissue is shown in Figures 1(c) and 1(d). The multiple and intensive bands for advanced glycation adducts were detected in diabetic cardiac tissues from ZDF rats. The elevated level of AGEs was significantly decreased by the administration of 500 mg/kg body weight per day of KIOM-79 (*P* < 0.05). In addition, RAGE expression in untreated ZDF rats was significantly increased when compared to its expression in control ZL rats. However, KIOM-79 treatment at the higher concentration significantly reversed the enhanced RAGE expression in ZDF rats (*P* < 0.05) (Figure 2).

### 3.3. Effect on Accumulation of Collagen in Heart

**Figure 3:**
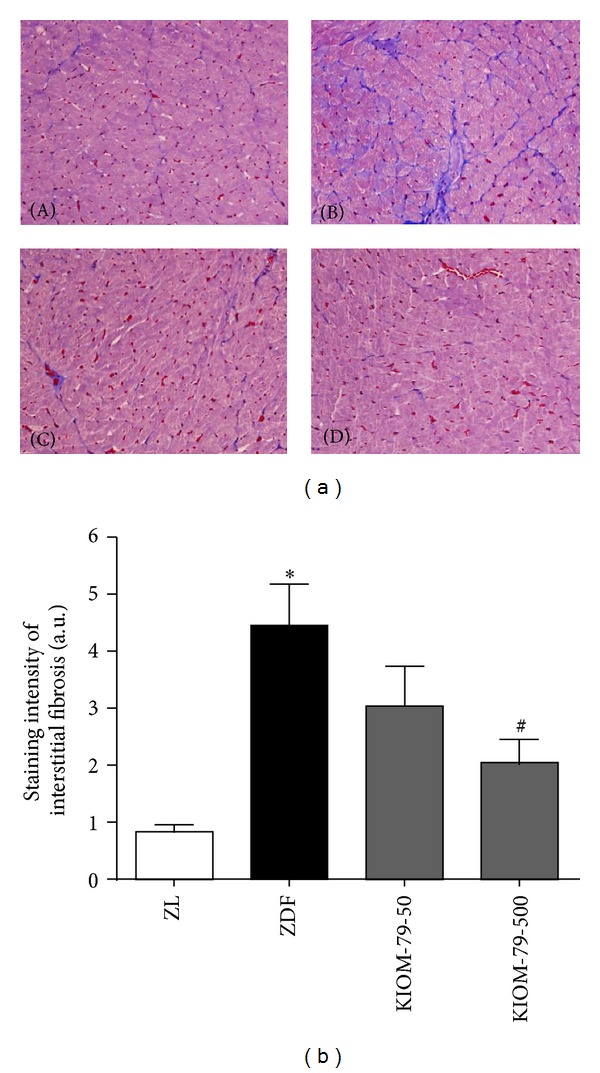
Effect of KIOM-79 on cardiac interstitial fibrosis. (a) Representative images for Masson's trichrome staining in the heart of ZL rats (A), untreated ZDF rats (B), ZDF rats treated with KIOM-79 (50 mg/kg) (C) and ZDF rats treated with KIOM-79 (500 mg/kg) (D). Positive staining is visible in blue. Original magnification: ×200. (b) Quantitative analysis. The positive stained area was calculated. All data were expressed as the mean ± S.E.M. *P < 0.05 compared to ZL rats; ^#^ *P* < 0.05 compared to untreated ZDF rats.

The degree of cardiac fibrosis was assessed using Masson's trichrome staining, and representative images are shown in Figure 3(a). Positive stained fibrotic area (blue color) was evaluated under a microscope. Collagen was detected in the interstitium of the heart of untreated ZDF rats at a greater level than in control ZL rats, and the collagen expression was lower in the heart of ZDF rats treated with KIOM-79 at the higher concentration.

### 3.4. Effect on Expression of Fibrogenic Factor in Heart

**Figure 4:**
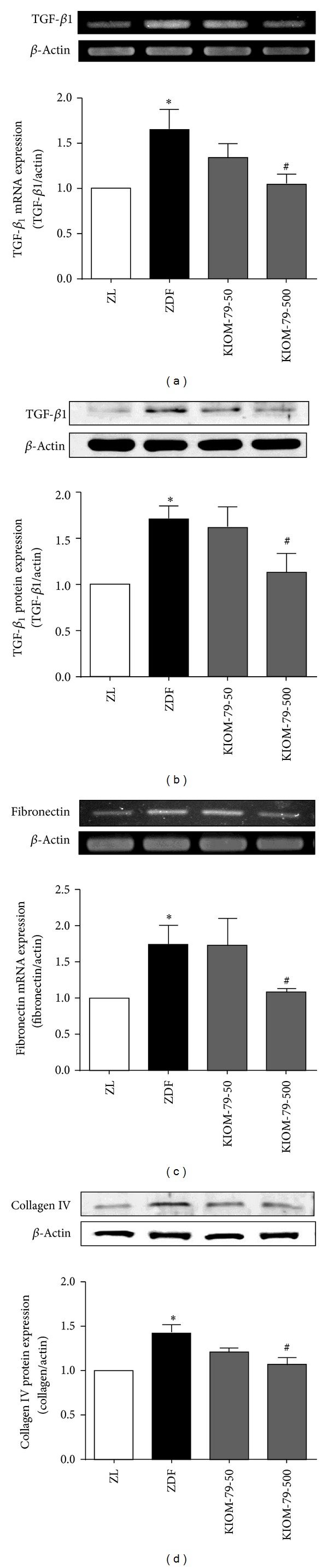
Effect of KIOM-79 treatment on the expression of TGF-*β*1, fibronectin, and collagen IV in the heart. (a) TGF-*β*1 mRNA expression, (b) TGF-*β*1 protein expression, (c) fibronectin mRNA expression, and (d) collagen IV protein expression. The results were normalized to *β*-actin. All data were expressed as the mean ± S.E.M. *P < 0.05 compared to ZL rats; ^#^ *P* < 0.05 compared to untreated ZDF rats.

Expression of a cardiac fibrosis factor, TGF-*β*1, was assessed by western blot and RT-PCR. The expression of TGF-*β*1 was found to be significantly higher in ZDF rats compared to control ZL rats (*P* < 0.05). High concentration KIOM-79 treatment in ZDF rats significantly inhibited the increase in TGF-*β*1 protein and gene expressions in ZDF rats (Figures 4(a) and 4(b)). In addition, fibronectin and collagen are major components of the extracellular matrix that play an important role in abnormal cardiac muscle function. Therefore, we examined their gene and protein expression in heart. Fibronectin gene and collagen IV protein expression were significantly increased in the heart of ZDF rats compared to control ZL rats (*P* < 0.05). Treatment with KIOM-79 at the higher concentration reduced the expressions of fibronectin gene and collagen IV protein when compared to control ZL rats (*P* < 0.05) (Figures 4(c) and 4(d)).

### 3.5. Effect on Expression of Urotensin II Gene in Heart

**Figure 5:**
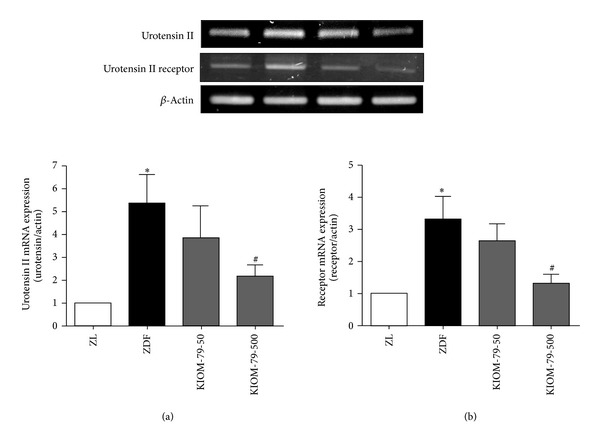
Effect of KIOM-79 treatment on the expressions of urotensin II and urotensin II receptor gene in the heart. (a) Urotensin II mRNA expression and (b) urotensin II receptor mRNA expression. The results were normalized to *β*-actin. All data were expressed as the mean ± S.E.M. *P < 0.05 compared to ZL rats; ^#^ *P* < 0.05 compared to untreated ZDF rats.

To investigate the molecular mechanism underlying the anti-fibrogenic effect of KIOM-79 in ZDF rats, we examined the cardiac expression of urotensin II and its receptor. Cardiac gene expression of urotensin II in ZDF rats was markedly increased compared to control ZL rats (*P* < 0.05). The treatment of KIOM-79 dose-dependently reduced the expression of urotensin II. In addition, the elevated urotensin II receptor level in untreated ZDF rats was also reduced with treatment of KIOM-79 in a dose-dependent manner (Figure 5).

## 4. Discussion

The present study demonstrates that KIOM-79 treatment decreased AGEs accumulation and RAGE expression in the heart without hypoglycemia. Although the levels of fibrogenic factors were unchanged with low concentrations of KIOM-79, mRNA and protein levels of fibronectin, collagen IV, and TGF-*β*1 were significantly reduced in cardiac tissues of ZDF rats treated with high concentrations of KIOM-79. Furthermore, KIOM-79 treatment reduced urotensin II and urotensin II receptor mRNA expression. These results suggest that KIOM-79 treatment may modulate AGEs accumulation and the urotensin pathway in cardiac tissues, which may play a role in reducing cardiac fibrosis in ZDF rats. 

KIOM-79 was developed based on the known ability of each herb to treat diabetes in traditional Korean medicine, and each herb also has anti-inflammatory, anti-AGEs, and antifibrotic effects [15–19]. In a 90-day repeated oral dose toxicity study in rats, the no observed adverse effect level (NOAEL) of KIOM-79 was at least 2000 mg/kg/day in both males and females (data not shown). Although the administration of KIOM-79 at 50 mg/kg body weight dose not significantly alter the level of AGEs formation and RAGE expression, our study showed that KIOM-79 dose-dependently inhibits AGEs accumulation and RAGE expression in cardiac tissues of ZDF rats. This finding agrees with the observation of our previous reports that showed that KIOM-79 has a strong inhibitory effect on AGEs formation and demonstrated beneficial effects of KIOM-79 on AGEs formation in the kidney [26] and retina [22, 23] of diabetic rats. Consistent with these studies, the present study shows that KIOM-79 inhibits AGEs accumulation in the heart of ZDF rats.

Prior studies have shown strong evidence for the positive role of AGEs in the process of the fibrogenesis [27–29]. In addition, some of the pathogenic effects of AGEs appear to be mediated by its interaction with AGE receptors [30]. TGF-*β*1 is a primary agent in fibrosis and the induction of TGF-*β*1 expression by AGEs results in collagen and fibronectin accumulation in the tissue, which is linked to the progression of diabetic complications including those observed in the heart [3, 6, 31]. In this study, treatment with KIOM-79 in ZDF rats suppressed the overexpression of cardiac TGF-*β*1, fibronectin, and collagen IV. Additionally, accumulation of collagen in the interstitium of the heart was ameliorated by KIOM-79 treatment. Our recently published study demonstrated that KIOM-79 inhibits expression of AGEs-induced TGF-*β*1 and fibronectin in a cultured mesangial cell line [25]. The results presented in this study confirm that KIOM-79 prevents the hyperglycemia-induced accumulation of AGEs and the expression of fibrogenic factors, such as TGF-*β*1, fibronectin, and collagen IV, in the cardiac tissue of ZDF rats.

Recently, a number of studies have suggested that the urotensin/urotensin receptor system may play an important role in the pathogenesis of diabetic cardiomyopathy. Expression of urotensin II and its receptor is localized to cardiomyocytes, endothelial cells, smooth muscle cells, and cardiac fibroblast of the diabetic hearts [12, 32]. Experimental and clinical studies have revealed an increased expression of urotensin II in animals with experimentally induced myocardial infarction and diabetes and in patients with diabetes [33, 34], as well as a promotion of cell proliferation and stimulated extra cellular matrix in diabetic animal models [8, 10, 35]. Consistent with these studies, the present study shows, for the first time, that urotensin II and its receptor gene expression are increased in the cardiac tissue of ZDF rats. Interestingly, KIOM-79 treatment dose-dependently inhibits the upregulation of urotensin II and its receptor gene expression in ZDF rats. These data suggest that KIOM-79 may regulate the cardiac urotensin pathway and may be partially involved in the regulation of profibrotic factor proteins.

Taken together, our finding demonstrate that KIOM-79 diminishes cardiac fibrosis in ZDF rats by preventing accumulation of AGEs and RAGE overexpression and by modulating the cardiac urotensin II/receptor pathway, which produces a decrease in profibrotic factors such as TGF-*β*1, fibronectin, and collagen in cardiac tissue. These data suggest that KIOM-79 may be a promising anti-fibrogenic agent in the diabetic heart and may delay diabetes-related cardiac complication caused by fibrosis.

## Figures and Tables

**Table 1:** Body weight, blood glucose, and heart weight at the end of the experiment.

Group	Body weight (g)	Blood glucose (mg/dL)	Heart weight (mg/100 g)
ZL	338.5 ± 40.5	92.93 ± 10.76	352.4 ± 11.4
ZDF	433.2 ± 69.4*	489.8 ± 038.0**	339.9 ± 14.1
KIOM-79-50	414.6 ± 45.0	391.70 ± 113.5	348.8 ± 20.9
KIOM-79-500	422.9 ± 49.1	390.80 ± 79.55	339.9 ± 15.3

All data were expressed as the mean ± S.E.M. *P < 0.05 compared to ZL rats; **P < 0.01  compared to ZL rats.

**Table 2:** Primer sequences for RT-PCR.

Gene	Sequence (5′-3′)
RAGE	5′-ACT ACC GAG TCC GAG TCT ACC A-3′ 5′-GCT CTG ACC GAA GCG TGA-3′
TGF-*β*1	5′-CGA GGT GAC CTG GGC ACC ATC CAT GAC-3′ 5′-CTG CTC CAC CTT GGG CTT GCG ACC CAC-3′
Fibronectin	5′-CAG GCT CAG CAA ATC GTG CA-3′ 5′-CCC CAC GAC CTA GGA AGT C-3′
Urotensin II	5′-TGC CTG CTC TTC GTA GGA CT-3′ 5′-AGA GCC TTC CTC AAG CTT-3′
Urotensin II receptor	5′-CTG TGA CTG AGC TGC CTG GTG AC-3′ 5′-GGT GGC TAT GAT GAA GGG AAT GC-3′
*β*-actin	5′-TCA TTG ACC TCA ACT ACA-3′ 5′-CAA AGT TGT CAT GGA TGA CC-3′
